# Extra Supplement.—The Nursing Mirror

**Published:** 1894-01-20

**Authors:** 


"The Hospital" Nursing Mirror.

Being the Extra Nursing Supplement of "The Hospital" Newspaper.

[Contributions for this Supplement should be addressed to the Editor, The Hospital, 428, Strand, London, W.C., and should have the word "Nursing" plainly written in left-hand top corner of the envelope.]

### News from the Nursing World.

### OUR PRINCESS.

In reply to numerous inquiries, we are glad to be able to state that the Princess of Wales is now convalescent, and we venture to hope that her visit to Brighton and cruise in the Mediterranean will completely restore her to health. Her Royal Highness's illness has been the cause of great solicitude, especially amongst nurses, for whom she has done so much, and has evoked widespread sympathy and devotion.

### A ROYAL EXAMPLE.

Presents of old linen have recently been sent by Her Majesty to many of the hospitals, and they have been thankfully received and gratefully acknowledged. The value of old linen is hardly sufficiently appreciated by those who have it in abundance, therefore nurses engaged in district work are constantly driven to make piteous appeals for worn underlinen, and clean, old white rags of every description for the service of the sick poor. Surely this would not be so if all good housekeepers would follow in the Queen's footsteps by sending at regular intervals consignments of old linen for the use of those who have none.

### DANGEROUS DECORATIONS.

"Yes, it's all very pretty, but I shan't have a moment's peace till the illuminations are over," was the quiet aside uttered by a hospital nurse at a recent entertainment. "You see, I know what burns are," she added, with an anxious glance along the gaily-coloured swinging lanterns. Thoughtless visitors and younger members of the staff laughed, chatted, and admired, but through it all the grave face of the woman who understood "burns" never relaxed its watchfulness. It is small wonder that the coroner's jury at Guilford condemned snapdragon as unsuitable for a hospital after the terrible tragedy which it recently caused, but many other so-called illuminations are little less dangerous to introduce amongst sick and helpless spectators.

### SAD REALITIES.

One of the prettiest and lightest wards at the Evelina Hospital is that which is set apart for children with whooping cough. Knowing the dismay caused to a nurse by the first sound of the unmistakable whoop, often emitted by a new patient suffering from another and perhaps serious ailment, the obvious advantages of a special ward are well-nigh unlimited. Two phrases are familiar enough in out-patient departments at all hospitals: "No beds vacant" is one, "case ineligible" is another. Both are as common as they are sad. It is only necessary to look at the disappointed friends of a rejected patient to realise the terrible gulf which lies between desirable and undesirable surroundings for the sick poor.

### WORK WITHOUT WAGES.

The present rules regarding the training of probationers at St. Bartholomew's Hospital—the richest hospital in the world—merit special attention from all who are interested in the welfare of nurses. Without personally perusing this wonderful new code, no one could credit its existence, mercy and justice being alike conspicuously absent. Nurse candidates, possessing all the high attributes demanded of the "ministering women" of the day, must engage to serve at this hospital for a term of four years. During this period each woman is nominally entitled to remuneration at a progressive rate, but the certainty of her receiving the pay is quite problematical, as the rules decree that a whole quarter's salary may be withheld if the probationer fails "to give satisfaction either to the matron or to the medical and surgical officers." It is not apparently necessary for a woman to be guilty of any serious fault, but if she does not "give satisfaction" to one of the selected officials, seemingly without right of appeal, the defenceless probationer may be fined to the extent of a whole quarter's earnings. The exceedingly precarious nature of the salaries secured by the St. Bartholomew's probationers is still further exemplified, for we read that sickness and sorrow can be punished as heavily as failure, it being also stated: "Nor will any part of a current quarter's payment be made to anyone who, from any cause whatever, leaves the hospital in the course of a quarter." Therefore the acute illness of a parent, the death of her nearest relative, or her own illness, may each be reckoned as a cause which by necessitating the nurse's temporary absence, will also subject her to the serious loss of a current quarter's payment. In case of her own death, the same confiscation of wages could doubtless be carried out, but as that detail would affect only her surviving friends, the consideration of it can be left to them. It hardly seems possible that those responsible for the government of a great charitable institution can be aware of the moral and legal liabilities which their probationers are asked to incur by binding themselves for four years to the service of a training school which obviously denies the existence of any rights onthe side of its workers.

### WITHOUT TRAINING OR EXPERIENCE.

Whilst many asylum managers are wisely ambitious to establish trained attendants under experienced supervision in their establishments, others seem markedly adverse to such progression. If this were not so, an advertisement would hardly set forth that no previous asylum experience is necessary in a lady superintendent who is required to be cheerful, musical, tall, energetic, and healthy!

### SIGNIFICANT SYMPTOMS.

"But, my daughter, how can you possibly teach your pupils to be methodical when you keep your own things in such disorder?" was asked by the tidy mother of a rather untidy schoolmistress. The daughter laughed as she answered, "It's easy enough to preach method, mother, and I keep all my own drawers locked." The young mistress merely put into words what a great many other folks practice. This is specially noteworthy at this season, as many of our readers must have observed whilst strolling through the various hospitals when they opened their doors to Christmas visitors. At some institutions bath-rooms, out-patients' departments, and the nurses' quarters have courted inspection, but at others, on the contrary, locked doors, or the placard "private" prevailed. These barriers, moreover, are not placed for the purpose of protecting the interests of nurses or patients, as an untrained philanthropist might charitably imagine. They invariably demonstrate that the nursing staff is badly lodged, and also that neither bed-rooms, bath-rooms, nor lavatories are fit to be shown. As in the case of the schoolmistress, practice and theory are widely apart.

### A PRECARIOUS LIVELIHOOD.

A nurse's career is recognised as being specially subject to changes. She has more opportunities than other women of contracting infectious diseases, and hence passing, by no fault of her own, through a period of weeks or months of enforced idleness. The well-established hospital worker is generally granted a reasonable amount of sick leave, which is certainly her just due, and few dare begrudge it to her. The private nurse is less fortunate, especially if she is "on her own account," and she should face this possibility of a short or long illness befalling her just as bravely as she does the other chances and changes of this mortal life. By reasonable care of present health and a conscientious provision for the inevitable days when it fails her, a woman, be she young or middle-aged, can secure ease of mind. When she has qualified herself for claiming sick pay from the Royal National Pension Fund, she can do her daily duty with that cheerfulness which is begotten of the knowledge that all possible emergencies are provided for.

### A NURSE'S RESPONSIBILITY.

The limits of a nurse's duties are not easily definable. Of course there is always an accepted code which deals generally with the subject, but the work for which a good nurse feels responsible combines a certain supervision of other people's duties. Perhaps this is specially true of the process of disinfection in private houses after illness is over. A woman of small experience is apt to think her duty fulfilled when she leaves her late patient's room ready for "the man" to fumigate. If "the man" happens to know his business things may go right; but if his knowledge consists in a few verbal directions from his master he forgets them promptly, because he does not comprehend their importance. So rooms are often fumigated and re-inhabited, and the household is dismayed, and the doctor puzzled by the eventual reappearance of disease. They are unaware that the window was imperfectly closed, or the chimney left open, and the quantity of sulphur insufficient. Perhaps the first patient was a child, and one of the family with misplaced thrift saved the toys and picture-books, which the nurse should have herself destroyed. Sometimes singed carpets and charred boards show that the fumigating apparatus was in most ignorant hands. A thousand unseen dangers lurk around the household where disinfection was nominal, not conscientious. All nurses should be accurately informed on this important subject, and should certainly never consider their duty accomplished until they have personally supervised all details of complete disinfection.

### VANCOUVER.

There are now six nurses attached to St. Luke's Home, Vancouver, including probationers who receive a certificate on the satisfactory completion of their two years' training. The Indian Hospital at Lyton is served by nurses from the Home, one of whom has resided there constantly since the opening in August. This arrangement will probably continue for another year. The St. Luke's Home has been considerably enlarged recently, and a convenient kitchen has replaced the old and inadequate on. An operating-room, another ward, two private rooms, bath-room, &c., have been added to the original building greatly to the advantage of patients and nursing staff.

### BENARES.

The care and attention received by the native patients in the Victoria Hospital at Benares are evidently appreciated. An old woman, acting as spokeswoman for many others, told an English worker there recently that "they come in tears, but go away laughing." The chief difficulty in carrying out medical treatment successfully in Indian hospitals arises from the restlessness of the sick. They have no patience to wait quietly for the effects of either internal or external remedies. The results of the treatment being not immediately visible, the patients are apt to grow home-sick and discontented. They crave to see miracles or charms in which they have hereditary confidence in spite of repeated disappointments.

### CUPBOARDS.

Surely a long and amusing essay might be written on cupboards—those convenient hiding-places of our ancestors, where the accumulation of years lay undisturbed, and where neither light nor ventilation were considered. Modern houses have seldom enough cupboards to please good managers, but those which exist are too often dark and suggestive of germ traps! The glass doors which are sometimes wisely adopted for medicine, splint, and other hospital cupboards, might with great advantage become more general. Steam kettles kept behind glass doors are certain to be in good condition for immediate service, and the same holds good of most things exposed to light and protected from dust. Even cupboards for which transparent doors are unadvisable should always be well lighted and freely ventilated.

### SHORT ITEMS.

The Union Guardians of Lewisham have courteously acknowledged and declined the offer of the St. John the Divine Sisters to undertake the nursing of their new infirmary.—Nurse Coulton, one of the staff of the Courtenay House Nursing Homes, Shepherd's Bush and Broadstairs, has been appointed by the Northampton County Council to give a course of nursing lectures.—Nurse Hassell has done excellent work amongst the sick poor since her appointment in 1889 to Crediton Sick Nurse Association.—Anyone interested in the work of the Mildmay Mission Hospital should pay it a visit. It is very near to Shoreditch Church, and can be reached by rail, tram, or omnibus.

### On Nursing the Nervous and Insane.

By T. DUNCAN GREENLEES, M.B.Edin., Medical Superintendent, Grahams Town Asylum, South Africa.

### II.—DISEASES OF THE NERVOUS SYSTEM.

It will suffice now perhaps merely to briefly note several of the more important symptoms that characterise disease or injury when the nervous system is implicated. Firstly, the organic forms (*i.e.*, where there is an alteration in the structure or a destruction of the tissues), I may refer to those diseases attacking the brain itself; and, secondly, to those affecting the spinal cord alone. Of the former class the best example is to be found in apoplexy, where a vessel ruptures within the brain, and the blood is effused into the tissues. According to the locale or position of the rupture paralysis of one or other series of muscles results, or consciousnes is lost. Should the rupture take place low down in the brain, and implicate one or other of the vital centres, then death takes place immediately.

In ordinary apoplexy the person usually loses consciousness suddenly, and drops down without any warning, the breathing is laboured, and at each expiration the cheeks are puffed out, the pupils are unequal, the conjunctivæ insensitive, and some paralysis of the legs or arms is generally met with. This paralysis is usually one-sided, and on the paralysed side pricking the skin meets with no response, showing the loss of sensation. Should the effused blood become absorbed, then the paralysis may pass off, but only provided the brain tissue has not been destroyed.

In the case of disease of the spinal cord, the paralysis usually attacks both sides equally, and should the disease be high up in the cord so as to implicate the inerves supplying the arms, the arms are likewise paralysed; this condition is, however, hardly consistent with life.

The most frequent form of disease found in the spinal cord is a sclerosis or hardening of the tissues following usually upon an inflammatory process, the result of chill or specific disease. Here the nerve tissues are replaced by a sclerosed patch, and the functions of all the nerves below the spot are obliterated. As a result of this, neural nourishment is cut off from the muscles, these become gradually paralysed, and atrophy or wasting sets in; the patient becomes bed-ridden; bed-sores tend to form, and, in the majority of cases, death closes the unhappy scene.

When disease attacks an individual nerve, all the muscles supplied by that nerve are paralysed, should the disease (or injury) be such as to prevent nerve impulses passing from the brain to the muscle or muscles.

Of the various forms of functional disorder of the nervous system the best example is hysteria. A "functional disease," or more correctly "disorder," exists where no appreciable organic disease can be discovered, when no change has occurred in the structure of the organ. As knowledge of nervous diseases increases the number of "functional disorders" diminishes, and scientific means, such as electricity, chemistry, and the microscope—by throwing light on hitherto dark and obscure questions—are proving that even in a "functional disorder" some change, of a morbid character, occurs in the molecules forming the cell or fibre. Hence the term "functional disease" must be taken provisionally.

Hysteria is a well known condition of the mental and nervous systems. Common in females of a certain age, it is not unknown in the stronger sex. It is characterised by symptoms of a most varied kind; from slight emotional feeling to a condition of intense excitement; from an apparently simple fit to what would seem, to the uninitiated, a most serious apoplectic seizure, together with innumerable intermediate conditions. Nevertheless its diagnosis is not difficult when taken in consideration with the age of the person, a probable previous history of hysteria, and on closer examination, entire absence of all those physical symptoms which indicate total and absolute loss of consciousness. In nine cases out of ten its diagnosis is easy, but often in the tenth case even the doctor mistakes a grave organic cerebral or spinal disease for hysteria, and in such a case the result does not reflect much credit on his professional acumen.

In the borderland between functional disorders and true organic disease many forms of hystero-epileptic conditions may be classified. Here the seizures are not so severe, nor are they followed by so marked mental disturbance as they are in ordinary epilepsy, but there is no sharp line of demarcation to be drawn between the two conditions, and one not infrequently merges into the other.

### TREATMENT OF NERVOUS DISEASES.

The advances that have been made during recent years in the treatment of nervous diseases can only be compared to the progress made in their pathology. Many of the means employed in the treatment are, however, still within the sphere of empiricism; it will be some years yet before the following methods will all be made use of by every general practitioner, and before his consulting-room will be replete with all the instruments necessary to the diagnosis and treatment of nerve diseases. When a medical man does undertake the treatment of such cases it is of the utmost necessity that his nurse, who ought to be his right hand as an assistant, should possess such a knowledge of the methods employed as will enable her to prove herself a useful and intelligent help to her employer.

*a.* ELECTRICITY.—The three forms of electricity most frequently used in medicine are Faradism, Galvanism, and Franklinism.

1. *Faradism.*—The machine consists of a cell and double coil, and this is the battery most commonly used, unless a large number of cells—and consequently a stronger current—is required. The Faradic current, generated within the cell, is conserved in the coil, and is carried thence, when in use, along insulated wires.

2. *Galvanism.*—Here the current is derived directly from the cells. Any two dissimilar substances, such as zinc and carbon, if immersed in an acid or saline fluid and then brought in contact, generate electricity, and this constitutes a battery of one cell. Galvanic batteries generally contain a number of these cells, whose forces can be united to produce a powerful current, or only one cell may be used when a weak current is desired.

2. *Franklinism* is obtained from the friction of two discs of glass, the one stationary, the other revolving rapidly on its own axis. In this form of electricity a flash of light is generated, and ozone, a peculiar transformed condition of the atmosphere, is appreciable to the sense of smell. This form of electricity is rarely used in medicine, and the machine now is chiefly used in laboratories for the purpose of demonstrations.

### Christmas in Paris.

A BEAUTIFUL Christmas tree was displayed on December 30th at Miss Christie's Orphanage, 54, Rue Violet, Grenelle. The Marchioness of Dufferin and Ava presided on the occasion, accompanied by the Rev. D. Noyes and the Rev. M. Makey. The children sang some charming French and English hymns, after which prizes and presents were distributed by Lady Dufferin. The gifts were sent by a number of English and American ladies, and flowers from Nice and dried fruits from Jerusalem were also contributed. The orphanage contains 19 children, about half being of British birth.

### District Nursing.

### III.—PRACTICAL WORK.

### The Uniform.

The first details to be considered in beginning the actual work are the uniform and the bag. The uniform must be practical and durable.

The dress should always be of washing material, ordinary walking length, and plainly made, as frills and pleatings only harbour dust, and, alas ! vermin also. Large bib aprons without pockets, and oversleeves buttoning closely at the wrist and coming over the elbow are absolutely necessary. These are better made of brown holland for town wear at any rate, as white aprons are a consideration with the really dirty work that has to be done, and a nurse at work in a soiled apron and sleeves is most unprofessional. Pockets are very undesirable, as they may form an unsuspected lurking place for germs, and in going from case to case a nurse must be most careful in this respect. For the same reason, no rings, except a plain gold band, or jewellery of any kind beyond a plain watch chain, should be worn while on duty. The sleeves protect the dress sleeves, and fastening at the wrist is a necessary precaution against the enemy !

Two cloaks are necessary for summer and winter wear. The summer one should be thin and yet shower proof; the winter one as warm and yet as light as possible. District nurses cannot bear thick, heavy materials—in wet weather they become heavier still, and are a great addition to the fatigue of walking on muddy roads or slippery streets.

The shape should be one that distributes the weight equally and allows the arms to be put inside or out with perfect ease. Circular cloaks are not sufficient protection when it is necessary to hold up an umbrella. The district cloak should have conveniently-placed arm-holes, and be of sufficient width to button easily all down the front, and yet allow plenty of room for the bag to be carried underneath. If carried outside the bag soon becomes shabby, and is apt to rub the cloak also. Adjustable hoods are a great convenience to country nurses, as they can be drawn right over the bonnet, and so dispense with an umbrella—a consideration to those whose distances require a pony cart. Plain, broad-brimmed hats are also desirable for these nurses, whose duty requires them often to drive some miles along glaring, dusty roads in the height of summer. For general wear, a neat bonnet, trimmed with ribbon in preference to velvet, in case of sudden showers, with a white frill and white washing strings is the most suitable.

Veils are a mistake for district nurses; they quickly become shabby, and are in the way of the work. They look untidy also when tucked into the apron straps at the back, as is often done of necessity when the nurse is stooping over the beds, &c. Special care should be paid to the boots and shoes. Heavy ones are bad for the feet, and increase the fatigue of walking.

Light, strong soles, soft leather, square toes, and broad, moderately high heels, are the most suitable.

Woollen underclothing should always be worn, even in summer, for the nurse is easily liable to chills when tired and overheated with her work.

Gloves ought not to be dispensed with in winter, as the skin is tender with the use of so much water, as well as the various liniments, &c., and cracks and roughnesses predispose to poisoned fingers.

### The Nurse's Bag.

The next consideration is the bag. It must be as light as possible, and yet large enough to carry all the nurse requires. The one made for the Queen Victoria's Jubilee Institute by Houghton and Sons, Southampton Row, W.C., is the best hitherto, fulfilling both these essentials. It is lined with leather, has a stand for six 1-ounce glass stoppered bottles, and six glass pots, 1½-ounce size, with screw lids, two inside pockets, and one outside. Five of the bottles are plain glass, and contain methylated spirits (for backs), turpentine, olive oil, carbolised oil, creoline. The sixth is coloured, and contains pure carbolic acid. The pots contain powder for backs (1. boracic to 2. starch), boracic powder, permanganate of potash, boracic ointment, zinc ointment, carbolised vaseline. A small pot of iodoform is also carried, and corrosive sublimate tabloids in special cases. A medicine and minim glass in case, a complete Higginson syringe, glass tube (without a terminal hole), and flexible catheter in a sponge bag, a 2-ounce glass syringe, a poultice spatula, a light tin case for strapping, an ink bottle, box of matches, and needlecase, with thimble, bodkin, tapes, cottons, and safety pins are all needed.

### The Nurse's Case.

Each nurse usually has her own case, which contains a clinical thermometer, probe, surgical and nail scissors, dressing forceps, caustic holder, and ointment spatula. If the nurse has not a second hand on her watch, a 30-second pulse glass is necessary. A square of thin mackintosh is advisable, on which dressings can be prepared, and which can be used to cover a fomentation in an emergency. A light papier-maché receiver is also needed. A washing bag shaped like a nightdress case divided into four contains old linen, wadding, tow, and carbolised tow for ordinary use. Special dressings, such as medicated lints and wools, should be securely wrapped in thin mackintosh or jaconet. The inner pockets contain pen and pencil, blotting paper, and the charts, and message papers for the doctors and the envelopes for them. The outer pocket contains a small hand-towel, and soap box with carbolic soap and nail brush.

Other nursing appliances, such as bath thermometers, ball syringes, &c., are usually provided, but only taken for special cases. The great point is to carry all essentials, but no extras.

### The Departure.

Thus provided with uniform and bag, the nurse is ready to start on her round; but, as may be imagined, her first feeling, when introduced to her work, is utter bewilderment. Everything is so totally different that though there are both patient and doctor's orders, she does not know how to begin. The case is probably one of pneumonia sent by a doctor, with orders for jacket poultices, temperature, &c.; the patient a carman, about thirty-five, with wife and three or four children, in a tidy but not too well-furnished home. They may have one fair-sized front room and a small one at the back, or perhaps only the one, depending on the rents of the locality. To the seasoned district nurse it is an oft-told tale, and at a glance she takes in the possibilities of the position. As the nurse enters she greets both patient and wife, and explains the doctor has sent the nurse to help the latter. It should be remembered the wife often rather resents her husband being cared for by others, and it needs a little tact to make her feel she is not being supplanted. The cloaks are removed and placed out of the way on a chair, clothes'-line, or behind the door, never on or near the bed or sofa if the nurse be wise !

### The Nurse's Duty.

The temperature, pulse, and respirations are first recorded, and the nurse ascertains a little of the history of the disease and what has already been done. The necessary things are then hunted up, basin, poultice basin, comb, two towels or substitutes, flannel and soap, rag and linseed for the poultices, and a kettle of boiling water. A piece of tow, back powder, and methylated spirits are also put ready. Unless sponging has been ordered it is wiser not to attempt too much the first visit—washing the patient's face, neck, and hands is enough. It is better not to move the shirt and vest either unless unavoidable, as the nurse can notice the patient's size and bring a proper pneumonia jacket on her next visit. The back is then attended to, clean sheet put in after shaking up the bed a little, and poultice applied with plenty of wadding as patient lies on his side. Turning him on his back again the chest poultice is put on, and fastened at shoulders and sides with safety pins. The top sheet, which has been hanging by the fire, is replaced, the blankets turned, the quilt spread, and the pillows arranged to suit the patient. It is well to give nourishment during this work and after it is done. The nurse enters the particulars of the case as to age, &c., in her note-book for the register of cases, and ascertains the symptoms, which she reports to the doctor on the printed memorandum form, leaving it with the chart for him. A few suggestions as to placing the bed in a better position because of draughts, the removal of soiled clothes from under the bed, the arranging a shawl or blanket to exclude draughts if the bed cannot be moved may be done, but it is wiser to make alterations gradually, and put both patient and room into nursing order by degrees.

The nurse washes her hands, and having cleared away poultice basin, and emptied dirty water, &c., leaves for the time. She first writes out a time table for the regular giving of medicine, stimulants (if ordered), and nourishment, for the guidance of the friends.

A SECOND VISIT.

At the second visit the shirt can be removed, more thorough ablutions done, and the pneumonia jacket put on with the poultices, and clean bed linen if needed. The patient is more accustomed to the nurse, and the wife has more confidence in her. Sponging should never be attempted for any cases without definite orders from the doctor. This is one of the great difficulties of district work, the absence of definite rules as to the nursing, and the difference of treatment amongst the doctors.

EXPERIENCE OBVIOUSLY NECESSARY.

This is another reason why great experience is needed, to distinguish what every case needs individually, and to be able to adopt fresh lines of treatment readily. There are some general rules, of course, but very few. It is unsafe to give any fixed ones, even for washing and making of beds, as the rules are considered before the patients, who are apt to sacrificed to the zeal of the nurse for "nursing order." Cleanliness can, and must be, brought about, but often it is a question of time and tact too. Patients often object strongly to washing, and their pitiful accounts frequently induce the doctors to order as little as possible, for not knowing the guarded way in which it is done they are led to believe the process much more trying than it is.

The rearrangement of the rooms must depend on the nature of the case, and also on the amount of furniture. Each case is a fresh lesson in itself, and no matter how long a nurse continues in the work, new experiences await her.

### Minor Appointments.

CUCKFIELD UNION HOSPITAL.—Miss E. R. Turner has been appointed Head Nurse at the Union Hospital. She was trained at the Norfolk and Norwich Hospital, held the position of Charge Nurse at the Royal National Hospital for Consumption at Ventnor, and was Charge Nurse at the Western Fever Hospital at Fulham. We wish her success in the responsible post which she has been chosen to fill.

CITY HOSPITAL SOUTH, GRAFTON STREET, LIVERPOOL.—Miss Honora Frances Cronin has been made Charge Nurse at this hospital. She was trained at Bradford Infirmary and Leeds Fever Hospital. Miss Cronin did private nursing in connection with the Bradford Nurses' Institute, was Charge Nurse at Dewsbury General Infirmary, and on the staff of the Leeds District Nursing Association. We wish her every success.

### Canadian Notes.

(FROM OUR TORONTO CORRESPONDENT.)

PEOPLE here are suffering from the prevailing depression, and as a result the hospitals lack pecuniary support at the present time to an extent which is becoming serious.

The Toronto General Hospital continues to flourish under Dr. O'Reilly's able superintendence, and some new buildings are in course of erection. They include a new instrument room, which will form quite a feature in the institution when completed, and a surgeon's lavatory. These additions have been placed near the operating theatre.

For eighteen years Dr. O'Reilly has managed the Toronto General Hospital. During that period the system of nursing has been entirely remodelled, more trained nurses have gone forth from this institution than any other in Canada. No one who has inspected the hospital and seen the nurses at work can fail to be impressed with the superior character and ability of the graduates of this school, many of whom are holding important positions in the large hospitals of the United States at the present time.

Great credit is due to Miss M. A. Snively, the Lady Superintendent, whose account of the work of the Nurses' Training School appeared in THE HOSPITAL about two years ago. A further account will doubtless soon be contributed by her, and a report of the work of the school for the past year. Miss Snively has a staff of 60 nurses, that is about one nurse for every six of the 350 beds the hospital contains, of which about 250 are constantly occupied throughout the year. The Training School for Nurses was established in 1881, and the system of training is excellent, the examinations being most practical and thorough. At the present time only two years' training is compulsory, but the authorities are thinking of instituting the three years' course, so as to bring their certificate into line with the highest standard.

The opening of the Royal Victoria Hospital at Montreal by the Governor-General, Lord Aberdeen, who was accompanied by Lady Aberdeen, created great interest in Canada, and all sections of society and public opinion were represented on the occasion. The Victoria is a fine hospital, an account of which appeared in THE HOSPITAL for July 29th, 1893, p. 285, and the appointment of so able and experienced a lady as Miss Draper to the post of Lady Superintendent is a guarantee that the Nurses' Training School will be placed upon a sound footing from the commencement. The hospital is replete with every modern appliance, the decorative part has been effectively carried out, and the wards are most satisfactory in all respects.

Dr. O'Reilly has just returned to Toronto after making an inspection of the chief infectious hospitals in the United States, with the view of preparing a report for the information and guidance of the health authorities in Toronto. For some time past an animated debate has been proceeding on the question of the advisability of maintaining a separate hospital for infectious disease and small-pox apart altogether from the Toronto General Hospital. The best solution of the problem will no doubt be for the municipal authorities to build and maintain an infectious hospital, and for them to contract with Dr. O'Reilly to undertake the nursing at a fixed rate per annum. It is not easy to estimate what the payment should be, but an annual fee for the work has been suggested with a scale of remuneration per month for each nurse supplied, beyond a certain minimum number, based upon the number of fever cases which it has been found necessary to provide for during the last three years. If Dr. O'Reilly is charged with the duty of providing the nurses, the public will have a guarantee that the work will be most efficiently carried out, and that the fever hospital will accomplish a maximum of good, because it will thus secure the confidence and support of all classes of the community.

### Nursing in Paris.

### L'HOSPICE DE LA MATERNITE.

A new wing which was recently opened at the institution known as L'Hospice de la Maternité at Paris, is designed for prematurely born as well as sick and infirm children. Many friends were invited for the occasion, and they were received in the clean and modest parlour of the establishment, which was prettily decorated with palms and chrysanthemums of all colours, which gave a very bright effect to the scene.

The president at this interesting ceremony was Monsieur Tarnier, the doctor of the hospital; at his side were placed M. Pegron, Director to L'Assistance Publique, M. Straus, Muncipal Councillor, Dr. Pinard, and many other celebrated doctors. With cordial, well-chosen words the President thanked all present for their help and devotion to the hospital, and particularly mentioned Madame Henri, the Matron, who, together with Dr. Tarnier, had perfected the "Couveuse." In its original form it was only a deal box of very imperfect construction, but Madame Henri dreamed of something better, and her dream was realised to-day. "Couveuses" at the maternity by fine boxes in glass, which are kept together by bronze bands and stand on four feet. They are in every way easy to keep clean. Madame H. explained, in a voice full of sympathy, the improvements in them. In the old kind the temperature was not kept up, neither could they be clean, and this was bad for the new born babies. By the old system only 10 per cent. of those prematurely born were saved. By the Tarnier invention a proportion of 33 per cent. lived, and since the last inovation introduced by Madame Henri the number of infants who survived was nearly doubled.

In the new wing overlooking the Boulevard of Port Royal (which has cost over 69,000 francs) Madame Henri was present, and she kindly answered all questions. Nothing could be odder, nor at the same time more touching, than the scene which met the eyes of the visitors in the first dormitory. On the right stood a line of little iron beds, with eider-down in pink and white, in which lay the children who had been prematurely born, but were now robust and enjoying their little lives like any other babies. Lifting the muslin which keeps away all draughts, quite a soft movement was visible under the quilt, and there lay the baby with eyes half open as if to welcome its visitor. Then a little cry, which was repeated by the wee neighbours as if to prove that all were alive and well. On the left of the room there stood about twenty of the glass cages looking like small aquariums, where little ones arrived too soon in the world were covered up until the due time arrived for them to breathe the ordinary air. One of these little creatures particularly attracted our attention by his fairness. He was born at six and a half months, and seemed to be doing well. His long white flannel had a pale blue border, and his little jacket ("brassière") was of the same colour, giving him the appearance of a pretty wax doll. All the babies were dressed alike, and hearty thanks were bestowed upon the generous donor of the pretty costumes.

Hot water pipes keep the temperature at 33 degrees C., and balls placed under the mattress of the "conveuse" are renewed three times in twenty-four hours. On the wall by each child there is a small card with its name, on which its weight is recorded each day, also its temperature and other little details. Next comes the "changing-room," where every five hours the children are "changed" on little cushions, without any unnecessary movements, and no trouble to the wee babies. In this chamber there were a dozen young women wearing grey with white caps, who replied pleasantly to the visitors' questions. They are the wet nurses, and beside each is her own child, big and fat and bright-eyed, some even running about. All look happy; when a bell rings the women go to the next room to their foster children, whom they feed every three hours. A room as big as the first we entered contains larger beds for the mothers, and wee nests beside them receive the new babies. The lavatory, dining-room, and garden were all inspected, and a little carriage, in which the babies go out in fine weather, stood in a shed, and near by the well-kept donkey who draws it was comfortably housed. Everybody and everything was sheltered in the "maternité." Happy children plucked from death become brave soldiers, good mothers, the glory of their country or the sacred guardians of home, and thanks are due to the good women who have accomplished so big a work. "To live is the right of all" is asserted on all sides to-day, and this doctrine is practically brought before us by those who established on so satisfactory a basis the Hospice de la Maternité. A. W. W.

### Women's Work.

### THE RESPONSIBILITIES OF MANUFACTURERS' WIVES.

The sanitary condition of the rooms in which female "factory hands" work, and the unsatisfactory meals which the girls and women partake of, merit all the attention which is beginning to be bestowed on these two most important matters.

The well-being of their husbands, their children, and themselves depends largely on these two factors, and therefore due consideration of them is claimed from all thoughtful women, as well as much practical help.

The recommendation made by Miss Bulley in her able article in the *Fortnightly Review*, that the wives and daughters of manufacturers might becomingly assume the duties of inspectors of the accommodation provided for the "factory hands" is worthy of attention.

When the wife is the true helpmeet of her husband she probably desires to receive no instruction as to her duty towards the women who work for "the master." She looks after them in sickness, and acts Lady Bountiful at weddings, christenings, and perhaps at funerals too. And in the present day she does more; she follows the fashion by organising lectures and distributing health tracts. But the ideal manufacturer's wife needs to qualify herself for her influential and responsible position. From her more should be demanded than a dole of beef-tea or a leaflet, although these are amongst the worthy means which lead to good ends. She should be able to show the women, and especially the girls, how to make nourishing broth or tasty soup. She should know why the sick room becomes "stuffy," and how this can be avoided without the draughts to which poor people, chiefly because they are ill-clothed and ill-nourished, have such a natural objection.

If sanitation has not been included in the education of a manufacturer's wife, let her bravely and modestly study the subject under competent guidance, and then let her teach it. To plunge into technical books without preliminary instruction is akin to the folly of sowing Bibles broadcast amongst heathen who have not grasped the first principles of Christianity. In exceptional circumstances good may result in both cases, but the world is made up of ordinary people and not of abnormally gifted ones.

The employer's wife and daughters will never become popular inspectors, but rather will be regarded as inquisitors if they act as mere fine ladies, dabbling in philanthropy. They must first understand thoroughly what is wanted, then how it can best be provided, and finally with full sympathy for the difficulties, poverty, and ignorance of their working sisters they must hold out to them strong hands of comprehensive fellowship, guiding them along the road which leads to the higher mental and physical life which is their uninherited right.

Miss Bulley divides the subject roughly into three heads —(1) The Wages Gained by Women Employed in Manual Labour; (2) Their Grievances; (3) The Effect of such Labour upon their Morality, their Home Life, and their Health.

Of course, all three sections hang more or less together, but first and foremost stands the influence of manual factory labour upon the health of women. It is regrettable that many factories leave much to be desired in the way of sanitation; nor are some of the large shops much better off. The right nail is hit on the head in the sentence: "What the women probably suffer from more, however, on the whole, than from the definite unhealthiness of any particular employment, is the lack of thoughtfulness in the minor particulars in their daily life, and in the small details which often make the difference between comfort and discomfort, health and ill-health." It is for "more kindliness, more consideration," that a plea is raised.

By women, nay, by women only, can more sweetness and light be brought into the lives of factory "hands"; but this cannot be accomplished in a hurry. Few manufacturers' daughters would be able to advise their fathers' employés as to how best to lay out their weekly wage to the greatest advantage in board, lodging, and clothing. Fewer still know how to make a simple and nourishing dish whose items should not be unduly costly. It is no use to tell a pale-faced girl that she is anæmic and must "feed herself up," when a daily meal of roast beef is as far out of her reach as champagne and turtle soup. She may listen to the well-meant counsel, but will go back to a meal of tea and white bread, with or without butter.

On the other hand there is practically no limit to the useful work which might be accomplished by a sensible woman, who, in a friendly manner (and without any suspicion of patronage) could practically disseminate the principles of cookery as well as of sanitation into the minds of the "hands." Her own knowledge need not necessarily be extensive, but it must be sound. She should be able to scour the saucepan properly before putting it on the fire to demon strate the making of a dish of stirabout; to prove, if need be, by immediate example, the precepts of her little chats on home cleanliness. She should have a thorough acquaintance with the properties of the various kinds of food-stuffs, and a better knowledge of vegetables than the average English cook possesses. Vegetables are never sufficiently utilised. Haricot beans, for instance, which are cheap enough, and which Sir Henry Thompson declares an excellent substitute for meat, would form a nourishing addition to eternal tea and bread and butter.

"It may be impossible," writes Miss Bulley, "to raise wages, but it should not be impossible to secure decent, healthy, and comfortable surroundings for the workers. It is probable that, were the latter once secured, the former would follow as a natural consequence. Women's labour is cheap now because it is, as a rule, inefficient; but every political economist knows the cheapest labour proves the dearest in the long run. A generation of better-nurtured women, with healthy surroundings, would become strong bodily and mentally, and would therefore be entitled to higher wages, for the efficiency of their labour would defray the increased cost of production. It is to be doubted whether, without increased efficiency, women's trade unions, to which so much faith is attached, can do much for the permanent advancement of the sex. The work of improvement must begin with the individual, not with the mass. Much may be done by placing before the working woman a higher standard of health and comfort, but to a certain extent—in the matter of wages at least—she must work out her own salvation, and fight her own way up. Those who are her friends should meanwhile assist in equipping her with the best weapons—viz., good health and common sense; sure that by so acting they are not only doing a kindness to their fellow creatures, but rendering an economic service to the nation.

C. H. H.

### The After-Care Association.

THE After-Care Association for Poor Female Convalescents on Leaving Asylums for the Insane held its annual meeting on January 15th, Sir B. W. Richardson being in the chair. The report for the year 1893 was read by the Secretary, and apologies for non-appearance from the Archbishop of Canterbury, Cardinal Vaughan, the Rev. H. C. Ridgway, Dr. Saunders, and others. The report was eminently satisfactory, the number of cases treated by the society being on the increase, but funds are needful for further extension of the work.

Speeches were made by Sir Benjamin Richardson, who explained the objects of the society, and Dr. Shuttleworth, of the Royal Albert Asylum, Lancaster, who emphasised the difficulty of obtaining employment for those discharged from asylums, the good work done by ladies in this direction being appreciatively mentioned.

Dr. E. W. White urged that the medical superintendents of asylums should be invited to co-operate with the society, and Dr. J. Kennedy Will spoke of the prejudice which existed against those who had been inmates of asylums.

Dr. Parker Young moved that the society should in future assist men as well as women, and its title, in consequence, be altered to "The After-Care Association for Poor Convalescents on Leaving Asylums for the Insane."

He was seconded by Dr. Rayner, who touched upon the subject of intemperance as a cause of insanity. He hoped their society would, at some future date, be in a position to deal with a question for which he urged immediate legislation.

Dr. Parker Young's resolution was unanimously adopted.

Dr. Hack Tuke, who was seconded by Mr. J. P. Richards, then proposed that the society should express its sympathy with kindred associations on the Continent, and he gave a short sketch of the work already done in Switzerland, where there are nine societies of the kind; in Italy, where it is proposed that the work hitherto done by voluntary associations shall be taken over by the State; in France, Germany, and Austria, where there are several such societies, supported partly by State subvention, partly by voluntary aid.

Among the other speakers were the Rev. W. St. Hill Bourne and Dr. Savage; and before the meeting broke up Mr. Sheen, one of the Poor Law Guardians for Kensington, expressed his opinion that the Board of Guardians should not be asked to assist the society out of the rates. One or two gentlemen present having expressed a different opinion, it was agreed to refer the matter to the committee. Sir B. Richardson, in his concluding speech, expressed his opinion that their society should not wait for State aid, which generally only arrived in time to put the seal upon work already accomplished.

### Death by Misadventure.

A NURSE has died at the London Fever Hospital, Liverpool Road, from the effects of narcotic poisoning. Apparently she unintentionally administered an overdose of laudanum to herself. On the very morning of her death the poor woman owned to having accustomed herself to opiates during the last two years. She promised the matron, who took much kindly interest in her, to take no more doses, and doubtless intended to keep her word. Unfortunately she failed to do so, and her life was forfeited by her own act within a few hours. Whether sleeplessness or pain originally caused indulgence in narcotics did not transpire at the inquest. In any case the unhappy event adds another to the tragedies which painfully show the prevalence of an indulgence in opiates by those who are perfectly acquainted with their dangerous properties.

### The Prevention of Consumption Amongst the Poor.

By Louis C. Parkes, M.D.

What is Consumption or Tubercular Disease?—In adults it is a destructive disease of the lungs, generally proving fatal within a few months or years of its onset. In children it is an affection of the abdominal glands, or of the membranes of the brain, or a disease of bones and joints. Children suffering from this disease are usually said to be in a decline.

Cause of Consumption or Tubercular Disease.—A microscopical germ or microbe, called the "bacillus of tubercule," which invades the bodies of those who are predisposed to take the disease. This bacillus is originally derived from the excretions of a person suffering from tubercular disease. The people who are predisposed to take the disease are those living under certain unhealthy conditions enumerated below, namely:—

1. Those who live in damp, dirty, or overcrowded houses or cottages, whether in towns or in country villages.

2. Men and women who, living in fairly healthy homes, are engaged for many hours of the day in overcrowded, heated, and ill-ventilated workrooms, more especially those whose occupations are sedentary, or which necessitate cramped and contracted attitudes, such as tailors, sempstresses, and dressmakers.

3. Men and women who work in shops or factories where the air is always dusty, very much heated, or very damp from the presence of steam. Breathing in dust particles into the lungs causes bronchitis, and issuing from hot and steamy air into cold raw winds causes lung inflammation. These constantly repeated lung troubles develop into consumption.

4. Those who from poverty or ignorance live on insufficient or improper food, more especially infants and young children.

5. People who lay too great a strain or tax on their vital powers, from over-work, anxiety, and exhaustion, and mothers who prolong lactation (the suckling of infants) beyond the ninth month after birth.

Prevention of Consumption or Tubercular Disease:—

1. Dirt aud overcrowding in dwelling-houses are more easily remedied than dampness of walls and floors arising from wetness of the soil under or around a house. Rooms which are very damp must be regarded as unfit for human habitation. Want of ventilation is especially injurious in bed-rooms.

2. Those engaged in sedentary occupations for many hours together should make a practice of spending one hour at least of the twenty-four in active exercise in the open air. Gymnastics, and open-air games which expand the chest, should be encouraged amongst the youth of both sexes.

3. Those who must work in very dusty atmospheres in factories should wear respirators when at work. Those who work in heated and steam-laden air should wrap up warmly before issuing out of doors, and on reaching home should sponge the body with tepid water to remove perspiration, and change their underclothing.

4. For adults the food should contain a fair amount of butter, dripping, or fat from meat, and the drinking of tea at every meal should be avoided. Infants, after being weaned, and young children should be largely fed on boiled fresh cow's milk. Condensed milks in tins and preserved infants' foods do not and cannot take the place of cow's milk. The milk should be boiled, as stall-fed dairy cows are very prone to suffer from "grapes," or tubercular disease, and the milk of such cows can infect children and produce tubercular disease in them. The high temperature of boiling destroys any contagion that may exist in the milk. The bottles used for feeding hand-fed infants should always be kept sweet and clean, and the arms and legs of all little children should be protected by clothing, and not exposed in the prevailing senseless fashion. After measles and whooping cough particular care should be taken to protect delicate children from chills.

5. Over-work, anxiety, and exhaustion should, as far as possible, be avoided; and mothers should be counselled to give up suckling as soon as the infant's first tooth is through.

6. Consumptive patients should be urged not to expectorate about rooms or streets, but to spit into rags which can be burned, or into cups which can be cleansed, their contents being disinfected and emptied into drains. No healthy person should sleep in the same bed, nor if possible in the same room, as a person advanced in consumption, and after death the room occupied by the patient, and all his clothes, should be thoroughly cleansed before being used again.

### Association of Asylum Workers.

Hon. Secretary, William Harding, M.D., Berrywood, Northampton.

It is proposed to start a society for the mutual benefit of men and women engaged in nursing the insane. The main objects of the association will be: (1) To raise the status of asylum nurses and attendants; (2) to help them to secure a system of training similar to that given to nurses in hospitals; and (3) to circulate amongst members a monthly journal called "Asylum News."

### Lecture at the Royal Free Hospital.

An interesting lecture, entitled "Relief of Lucknow, and other Engagements in the East," was recently given by General Gordon Pritchard, C.B., a member of the Committee of Management in the lecture room of the Royal Free Hospital, on Thursday last. It was fully illustrated by limelight views, and was much enjoyed by those of the patients, staff, and students who were able to attend.

### Appointments.

Ashby-de-la-Zouch and District Cottage Hospital.—Miss Harriet Raven has been appointed Matron of this hospital. She was trained at Tamworth Cottage Hospital, was Matron of Poole Cottage Hospital, and has held other responsible posts. We wish her all success.

County Asylum, Rainhill.—Miss Valentine has been appointed Matron of the County Asylum, Rainhill. She was trained at the Royal Infirmary, Wigan, where for some years she held the post of Senior Sister. She was then made Assistant Matron at the County Asylum, Lancaster. Miss Valentine takes with her the good wishes of many friends.

Home and Hospital for Jewish Incurables.—Miss Anstey, who has been Head Nurse at this hospital since it was opened in 1890, has been promoted to the post of Matron by the unanimous vote of the committee, by whom her services are gratefully appreciated.

St. Joseph's Children's Hospital, Temple Street, Dublin. — Miss Gertrude McGivney has been appointed Superintendent of Nurses at this hospital. Miss McGivney was trained at Sir Patrick Dun's Hospital, Dublin, and she has our best wishes in her new work.

Toxteth Park Workhouse Infirmary.—Miss Rebecca Ellis, who was trained at the Metropolitan Asylum, has been appointed Superintendent of Nurses at this infirmary.

### Presentation.

The nurses of the Eye, Ear, and Throat Hospital for Shropshire and Wales recently presented Miss Flemming, the Matron, with a silver cake knife.

### Wants and Workers.

A lady will be glad if any reader of The Hospital can tell her of a Home where a boy, slightly deficient in intellect, could be received. He is not eligible for an idiot asylum, and the parents are poor. A small payment can be guaranteed, and particulars learnt by writing to Miss E. R., care of the Manager, 428, Strand.

☞ For Nurses' Looking-Glass, Book Reviews, Everybody's Opinion, and Reading to the Sick, see page clix. et seq.

### The Muses' Looking Glass.

"THE PAINTER ART CRITIC."

MR. HARRY QUILTER'S EXHIBITION.

ABOUT half-past two on Saturday afternoon I found myself in front of the Egyptian *façade* in Piccadilly. I cannot say that its peculiar architecture reminded me cf any one of the many temples familiar to me on the banks of the Nile. Probably this was owing to its mundane western surroundings and the intermittent drizzle of damp misty weather. There was a considerable crowd of people outside the gates which reminded me of my early days when, as an enthusiastic playgoer, I waited patiently to pay my way into the pit of some favourite theatre. I knew that Mr. Harry Quilter was a popular man, and this jostling crowd, eager to see his first exhibition of pictures, did not surprise me. I found later when I had arrived as far as the Dudley Gallery that there were two shows and that the crowd were divided in their choice. Many went into the Home of Mystery on the right of the hall, but still Mr. Quilter's show of pictures attracted quite a number, for on entering the gallery I found it well patronised by well-known members of what is called smart society. The first person to attract my attention was a very graceful lady secretary, who presented me with a charming little catalogue in a scarlet cover, on which was printed "A catalogue of a first exhibition of pictures, sketches, and studies by Harry Quilter." The gallery was tastefully arranged after the fashion of a comfortable drawing-room, with tables, lounges, flowers, books, and bric-a-brac. The walls were festooned with drapery of chocolate hue. Neatly-dressed maids busied about the room serving tea and coffee. On one of the tables there was a suspicion of more potent beverages.

Unlike the ordinary picture gallery—the rooms absolutely walled with pictures in one confusing mass, which almost scares the visitor by the immensity of the task he sees before him—Mr. Quilter's pictures and sketches were arranged thoughtfully against the chocolate background, and were placed in frames, the hues of which were in sympathy with their subjects.

On looking at the pictures one feels that the well-known art critic justifies his position as critic of pictures and this is all. Mr. Quilter is no great painter but he knows enough to keep him in touch and sympathy with the art he so ably and justly criticises. In his first show of sketches and studies he proves that he is familiar with the various fashions of painting of to-day. There is the feeling of the impressionists, the realistic, and the old world schools throughout his one hundred and thirty-five studies.

There are landscapes, seascapes, and figures, both in water and oil. Figure painting is not Mr. Quilter's mission. In No. 1 "Rose Mary," though unfinished, the above fact is only too palpable. One is attracted at once from the main subject to the open window, and the landscape beyond, which is real good work. Land and seascapes are no doubt Mr. Quilter's *métier*. In "Bawdry Marshes," No. 38, we have a charming little picture. The rain-washed fields and rain-charged clouds are admirably painted. This small picture in an insignificant position is to me one of the best pieces of work in the exhibition. No. 112, "The First Rush of the Spring," takes its proper place if one stands a few feet in front of it. In fact, the majority of Mr. Quilter's works are so sketchy in the foreground, so slight in detail, that it is necessary before the whole work takes shape in many of them to look at the picture some six feet away. One of the few exceptions is probably No. 89, "The End of the Season." The picture is the most highly finished work in the exhibition. The sea is admirable, and full of that glow when the sun has dipped the horizon. "The Shipwreck Rottingden," No. 37, is a slight sketch, full of life. The crowd on the top of the sea wall, peering into the distant troubled waters is so suggestive of the impending disaster that one almost feels in sympathy with the folk anxiously watching the doomed ship. "The Lonely Shore," No. 116, is another example of an imperfectly worked out foreground. The middle and far distances in this picture are full of life and the tumble by the sea. One can see here the intention of the artist to focus the attention on the troubled waters by not painfully loading the foreground, but it is a little overdone. "The Work-a-day World" is charming. It gives the busy worker who may be overpressed a sense of peace and quietude so intense as to prove restorative. Very few more striking and attractive pictures than this have ever been exhibited.

"The Harbour of St. Ives," No. 68, is a bright little work, full of excellent colour.

No. 60, "The Miser's Orchard," is pleasing both in drawing and colour. The distant dip of the sea is full of sunlight and clean colour.

On the whole, the oil pictures have more merit in them than the water-colours, and one leaves Mr. Quilter's first show with the feeling that at present landscape is the branch of art he is most familiar with; that he has also a very happy knowledge of how to make a picture look well by its enframement. Some of the frames are exceedingly novel in design and good in tone. Altogether the show is a pleasant one, and interesting, especially to artists, for they can see what manner of man in the technical knowledge of art is the art critic.

V.

### Strange Consciences.

FOR a long while it has caused much anxious thought to people who know something of Indian life, to hear of English girls after two years' "preparation" going abroad and practising medicine. These are not the fully qualified medical missionaries who do valuable service in countries where men are not permitted to attend the female natives. Nor must they be confounded with trained certificated nurses, whose position is easily defined. These unqualified and partially trained lady helps are girls of limited means or limited patience, who do not submit themselves to the course of preparation, which alone justifies any man or woman in practising medicine. We have frequently alluded to the terrible dangers of such presumption. What would be thought in England or any other European country, if a medical student of either sex ventured to perform important operations? At last this most serious matter seems likely to get the attention which it demands, and eventually it is to be hoped that our sisters in other lands will be to some extent protected from unqualified medical missionaries as they are here, and also from uncertified nurses. The gentleman who wrote to the *British Medical Journal* last week in defence of these courageous unqualified practitioners evidently considers that the end justifies the means, knowledge ranking below enthusiasm. It is strange, indeed, to find an assertion in this connection that "With us it is entirely a question of conscience!"

### Royal Academy.

AT a general assembly of Royal Academicians, held on Tuesday at Burlington House, Mr. John M. Swan and Mr. Arthur Hacker, painters, were elected associates. No better selection could have been made. Mr. Swan holds a first place amongst animal painters. His work is always of the highest and best, and his devotion to art is widely recognised and appreciated everywhere. Mr. Arthur Hacker has been clearly marked out for this distinction, and his election will create no surprise. He is an industrious, zealous, and conscientious painter, whose talent has been readily admitted in art circles in recent years. He has accomplished much good work, but his youth, his talents, and his energy will no doubt combine to make his future productions more widely valued even than those which are already hung in some of the most representative public and private galleries.

### The Book World for Women and Nurses.

[We invite Correspondence, Criticism, Enquiries, and Notes on Books likely to interest Women and Nurses. Address, Editor, THE HOSPITAL (Nurses' Book World), 428, Strand, W.C.]

HOUSEHOLD NURSING. By JOHN OGLE TUNSTALL, M.D.Lond. (London: Fisher Unwin.)

"Household Nursing" is for trained nurses a very pleasantly written and readable little book, but can hardly be said to contain the rudiments of nursing suitable for the untrained, or anything really new for the trained. The advice given on general nursing is, however, quite worthy of being quoted. "From the medical aspect the nurse's function is twofold—to observe and report, and to carry out the doctor's instructions." . : . "The more a nurse knows the less will she presume, the better will she recognise that the doctor is some distance ahead of her in matters medical, and until she is qualified to sign a death certificate she will abstain from altering in any way his directions" (pp. 34, 35). "The best work is almost invariably done by those in the best health, and while it is a duty the nurse owes to herself, it is no loss to her patient if she so regulates her time as to maintain her own health" (p. 33).

All this is excellent, but we cannot help regretting that Dr. Tunstall should have written such directions as he has for the "untrained friend or member of the family in charge of the case."

For instance, he speaks of the (untrained) nurse giving antiseptic injections in parturition cases (p. 22) both before and after delivery, but there is no mention in any part of the book as to how such injections are to be administered, or what appliance is to be used for them.

In the chapter on Emergencies, and on Poisoning, the nurse is directed to give certain remedies by the bowel and not by the mouth, but no directions are given as to how this is to be done!

Nor is there any mention of the minor but very important duties of giving, removing, and emptying bed-pans, although at page 36 the use of a bed-pan is recommended in certain cases of disease.

Urinals, however, are not mentioned at all. Nor is there any mention of how the untrained woman is to change the personal linen of a patient while lying down without disturbing him; or to sponge or wash a patient without removing the bed coverings, *i.e.*, what is technically termed "sponging between blankets.

The necessity of having a trained nurse in cases of typhoid or diphtheria is strongly insisted upon, but one cannot help feeling surprised that the untrained nurse—always supposing that she has this book to help her—is assumed to be quite capable of nursing scarlet fever from the beginning to the end, with the mopping out of throat, &c., and disinfection needed.

Considering the grave nature of the disease, the serious evils that so often result from it, and the amount of skilled care necessary to prevent these arising, it seems a pity that the author should not have included it with typhoid and diphtheria.

Exception may also be taken to the directions with regard to excretions and discharges (pp. 40 and 52), where it is recommended that "discharges should be taken up on lint and burnt!" Why should lint, which is very expensive, be used, instead of bits of white cotton rag, or old linen?

Would it not also be more dangerous as well as very offensive "to keep all the excreta" of a typhoid patient "for at least twenty-four hours" before discharging them down the w.c.? Is it really an ascertained fact that no disinfectant has yet been discovered of sufficient strength to destroy the germs of typhoid upon contact?

Is it also generally admitted that *no* excreta need be disinfected except typhoid, as the author asserts?

On sanitary grounds would it not be better that all excreta should be received into some disinfectant before leaving the sick room? and if Dr. Tunstall thinks it unnecessary in scarlet fever, would he make no exception for cholera or dysentery?

The only new suggestion we can find in the book is at page 28, where it says: "A comfortable bed-rest may be formed by a long piece of netting, the ends of which are attached to the bed-foot, while the loop passes round the patient's back in the sitting posture." We have not seen this tried; but as few private houses possess a piece of netting long enough for such a purpose, it seems a pity not at least to have mentioned an inverted chair as a bed-rest—and a very good one—which is always obtainable.

The chapter on poisons and their remedies would be more valuable if presented in an abbreviated and tabular form. The author recommends that where carbolic acid has been administered, large quantities of warm water should be taken as an emetic, and when the stomach is nearly emptied, a solution of albumen should be administered to protect the surfaces of the viscera to be made by mixing the whites of three or four eggs with half a pint of cold water; or if there are no eggs at hand, to give one or two ounces of salad oil. Would it not be simpler to say give a wine-glassful of oil *at once* as an emetic before going for warm water? as it is a case where delay usually means death.

With regard to the few remarks on invalid diet it may be stated that no good cook, and certainly no trained nurse would "*oil* a mould" before filling it with blanc-mange, as it would spoil the flavour; and as certainly would not care to give any patient beef-tea which had been "strained clear from sediment." To quote from the official hand-book of the Royal School of Cookery*, "It is better *not to strain* beef-tea, as it removes all the little brown particles which are most nutritious."

"White wine whey" made with "concentrated essence of rennet" is a novel and round-about way of making it—a glass of white wine (*e.g.* sherry) added to a pint of boiling milk and allowed to stand for a minute by the side of the fire being simpler and pleasanter.

The object of a handbook on household nursing should be to enable an untrained mother or sister, in the absence of a trained nurse, to do the simpler acts of nursing for a sick relative. To keep her patient clean, to know how to change his linen without uncovering him or allowing him to sit up, to know the use of common nursing and sick-bed appliances, are of far more importance than to know how to take a temperature or apply a bandage. But these instructions have not been given.

* Lesson 15. Sick-room Cookery.

THE CENTURY MAGAZINE for this month devotes a number of its pages to a most eulogistic study of one of our most noted literary critics—Andrew Lang. The pen of the portrayer is American, and as Andrew Lang has been severe in the extreme in his criticism on American literature, the praises sung by an American brother must be regarded as very disinterested. The article is well worth reading, as the writer is intelligent and critical, as well as being a most evident admirer of Mr. Lang. He gives us in his short epitome of the critic's life and works ample opportunity of judging for ourselves as to how far we are in sympathy with Andrew Lang. Poetic, imaginative, and versatile as Mr. Lang shows himself in his works, as a critic he presents himself in another light. His opinions of American literature strike us as far too sweeping, and his wholesale condemnation of certain French writers suggests that even, wide reader as he doubtless is, that his acquaintance with their writings is too incomplete to warrant so harsh a judgment.

### Everybody's Opinion.

[Correspondence on all subjects is invited, but we cannot in any way be responsible for the opinions expressed by our correspondents. No communications can be entertained if the name and address of the correspondent is not given, or unless one side of the paper only be written on.]

### NURSING IN WORKHOUSE INFIRMARIES.

"ANOTHER WHO KNOWS," from experience in both a large workhouse infirmary (also a training school) and in a small infirmary where the nurses are few, can quite sympathise with the troubles described by "One Who Knows." In a large workhouse infirmary, such as the one at Birmingham, the nursing is conducted with as much thoroughness and *esprit de corps* as in our hospitals; in fact, there are many conveniences which smaller hospitals do not possess, whilst the conduct of other officials towards sisters and nurses leaves nothing to complain of; but in a small infirmary connected with, and under the same management as the workhouse itself, things are very different, and one meets with all those daily difficulties and frictions which are so small in themselves, but which make all the difference between the work being done satisfactorily, and therefore with pleasure to the nurse, and its being carried on in a way which a conscientious nurse cannot consider thorough, and therefore feels burdensome. The difficulties principally arise from there being no trained matron or real head of the nursing staff, and the number of patients under each nurse's charge; the only assistance, either by day or night, being given by inmates, who are seldom trustworthy and constantly being changed. A nurse in a small workhouse infirmary needs an unfailing supply of patience, cheerfulness, energy, and tact, there being none of the cheering and inspiriting influences from without that there are in a large institution. It is this principally that makes the work unsuitable to any except those of a very vigorous constitution mentally and bodily, whilst on the other hand, vigorous and energetic minds usually prefer the greater interest and excitement of hospital life or the change and variety to be found in district nursing. This may probably account for the difficulty in obtaining really efficient nurses for small workhouse infirmaries, though to capable women, who are not easily disheartened but are content to work and wait for results, the improvements which they will in time be able to bring about for the better nursing of those under their care will be sufficient reward.

### MATERIALS FOR UNIFORM.

NURSE OLIVE writes: Will someone kindly inform me if it is usual for hospital and infirmary nurses to pay for having their uniform dresses and aprons made? The hospital where I am requires that each nurse should either make or pay for the making herself.

*** At many hospitals the materials for the uniform are provided, but at very few is the cost of making defrayed. It is unreasonable to expect it, as is shown by the reflection that a woman engaged in any other way would naturally have to pay for materials as well as for making up all her clothes. When laundry expenses are defrayed by an institution which also provides "washing materials," the wearing of uniform becomes economical as well as suitable. The question as to whether all uniform is provided or only "a certain part" of it, should always be made plain to candidates before, not after, engagements are entered into.—ED. *T. H.*

### DISTRICT NURSING.

A "DISTRICT NURSE" writes: Allow me to express my sincere admiration of the articles at present appearing in THE HOSPITAL about "District Nursing." Having been a district nurse myself I know from experience how very true and to the point they are. District nursing is a subject still very imperfectly understood, and often wofully mismanaged by amateur meddling; therefore all nurses who love their work will thoroughly appreciate these clever articles.

### For Reading to the Sick.

### DUTY.

"The trivial round, the common task,
Will furnish all we ought to ask,
Room to deny ourselves, a road
To bring us daily nearer God."

MEN have two very different parts to play in their lives, one is the quiet, humdrum of every-day work, the other the rarer call, which however sometimes comes, to make a great effort of courage or self-sacrifice. Every man or woman goes on apparently very much the same, but when the opportunity arrives one person will act like a hero while another is a coward, a dead failure. I suppose it is the way in which we have spent our quiet hours which causes the difference in our behaviour at a crisis. One man has been going on quietly in the path of duty, doing what lies close at hand with a single eye, while another thinks only how he may get the best of everything for himself—riches, fame, glory—and though the love of fame and glory will sometimes lead to great deeds, they are not enough, even for this life, to carry men on through storm and sunshine—happy, peaceful, contented to the end. Our blessed Lord teaches us quite plainly and quite perfectly how to go on in this path of strength and peace in the words, "I came down not to do Mine own will but the will of Him who sent Me." He who died to set us free to live the life that men should live, shows us by His own example how our plainest tasks may be our training for the very noblest deeds. Not to do His own will but his Father's will was the central principle of His life. To be subject to His Parents in childhood and youth, to work honestly and conscientiously in a carpenter's shop in manhood, do not seem to us very great things, but they were the preparation for the most tremendous sacrifice the world has ever seen—for Christ's laying down His life for His enemies.

"Not to do mine own will," is the resolution which is needed, and by seeking God's grace to do the most ordinary tasks, the mere drudgery of life will be ennobled and shine with some reflexion of the obedience of Heaven. The constant disciplining of ourselves will cause us to take for granted that duty must at all times be to the front and that everything else must give way to it. "The love of duty is the strength of heroes," whether like our great soldier and sailor Wellington and Nelson, it is used for the love of country, or in the passive virtues of patience and endurance as shown by the Emperor Frederick.

Indeed, there is no time in which a life of duty stands us in better stead than in sickness. If we have always given way to our fancies in health, shirking everything that is unpleasant, or that we do not like, how shall we bear pains and anguish which are unavoidable? Duty was the keynote of the Emperor's life and his preparation for governing Germany. But God had a different plan, and the Emperor has left a great example of unmurmuring patience to teach and encourage men through all time and in all ages, to do their duty simply and quietly, even through the weariest days of suffering and weakness.

### Presentation of the Silver Cross.

MR. WILLIAM FAIRBANK, surgeon to Her Majesty's household at Windsor, has received the Silver Cross of the Order of St. John of Jerusalem, in recognition of his services in furthering the cause of the association by the establishment of railway and other ambulance classes for giving first aid to the injured.

### Where to Go.

TOYNBEE HALL, January 20th.—At eight p.m., Mr. Ernest Hart will give an address on "The Responsibilities of the Press, Especially in Respect to Quacks and Patent Medicines."

